# Cooperative Genomic Lesions in HRAS-Mutant Cancers Predict Resistance to Farnesyltransferase Inhibitors

**DOI:** 10.21203/rs.3.rs-3154719/v1

**Published:** 2023-07-14

**Authors:** Aradhya Nigam, Gnana Krishnamoorthy, Walid Chatila, Katherine Berman, Mahesh Saqcena, Henry Walch, Alan Ho, Nikolaus Schultz, James Fagin, Brian Untch

**Affiliations:** Memorial Sloan Kettering Cancer Center; Memorial Sloan Kettering Cancer Center; Memorial Sloan-Kettering Cancer Center; Memorial Sloan-Kettering Cancer Center; Memorial Sloan-Kettering Cancer Center; Memorial Sloan-Kettering Cancer Center; Memorial Sloan-Kettering Cancer Center; Center for Molecular Oncology, Memorial Sloan Kettering Cancer Center; Memorial Sloan Kettering Cancer Center; Memorial Sloan Kettering Cancer Center

## Abstract

The clinical development of farnesyltransferase inhibitors (FTI) for *HRAS*-mutant tumors showed mixed responses dependent on cancer type. Co-occurring mutations may affect response. We aimed to uncover cooperative genetic events specific to *HRAS*-mutant tumors and study their effect on FTI sensitivity. Using targeted sequencing data from MSK-IMPACT and DFCI-GENIE databases we identified co-mutations in *HRAS*- vs *KRAS*- and *NRAS*-mutant cancers. *HRAS*-mutant cancers had a higher frequency of co-altered mutations (48.8%) in MAPK, PI3K, or RTK pathways genes compared to *KRAS*- and *NRAS*-mutant cancers (41.4% and 38.4%, respectively; p < 0.05). Class 3 *BRAF, NF1, PTEN, and PIK3CA* mutations were more prevalent in *HRAS*-mutant lineages. To study the effect of comutations on FTI sensitivity, *Hras*^G13R^ was transfected into ‘RASless’ (*Kras*^lox/lox^;*Hras*^−/−^;*Nras*^−/−^) mouse embryonic fibroblasts (MEFs) which sensitized non-transfected MEFs to tipifarnib. Comutation in the form of *Pten* or *Nf1* deletion or *Pik3ca*^*H1047R*^ or *Braf*^*G466E*^ transduction led to relative resistance to tipifarnib in *Hras*^G13R^ MEFs in the presence or absence of Kras^WT^. Combined treatment of tipifarnib with MEK inhibition sensitized cells to tipifarnib, including in MEFs with PI3K pathway comutations. *HRAS*-mutant tumors demonstrate lineage demonstrate lineage-dependent MAPK/PI3K pathway alterations that confer relative resistance to tipifarnib. Combined FTI and MEK inhibition is a promising combination for *HRAS*-mutant tumors.

## Introduction

The global incidence of *HRAS*-mutant tumors is approximately 230,000 per year- representing 7% of all *RAS*-mutant tumors.(1) Activating mutations in the *HRAS* oncogene have been described with varying prevalence across multiple cancer lineages including bladder, head and neck squamous cell cancer (HNSCC), lung, thyroid and melanoma.(2) Targeting RAS-mutant tumors remains challenging. (3) Treatment approaches aimed at targeting upstream and downstream effectors have been met with mixed responses (4–7). The recent development of *KRAS*-G12C inhibitors and their consequent FDA approval for non-small cell lung cancer shows the benefits and limitations of directly inhibiting RAS drivers (8, 9), and highlights the importance of targeting other members of the RAS family.

Tipifarnib, a farnesyltransferase inhibitor (FTI), is a targeted therapy for *HRAS*-mutant tumors.(10–12) FTIs block the addition of the farnesyl lipid moiety on the C-terminus CAAX motif of *HRAS* and prevents its translocation and subsequent activation at the plasma membrane.(13, 14) Although originally developed as therapy for *KRAS*-mutant tumors, the discovery of rescue prenylation by geranylgeranyl transferase results in an alternative pathway for activation of KRAS and NRAS, but not *HRAS*.(15) A phase II trial evaluating HNSCC patients with *HRAS* mutations showed reduction in tumor burden and improved progression free survival, particularly in those with a high variant allelic frequency of the mutant oncoprotein.(16) In addition, pilot trials have demonstrated efficacy of tipifarnib in patients with metastatic salivary,(17) and urothelial cancers(18) with *HRAS* mutations. However, for unclear reasons, similar responses have not been reported for other cancer types, including thyroid.

We previously reported that murine (TPO-Cre/*HRAS*^*G12V*^/*TP53*^*flox/flox*^) poorly differentiated thyroid cancers developed acquired resistance to FTIs associated with mutations in *Nf1* and *Gnas*, which recapitulated FTI resistance when introduced into HRAS-mutant thyroid cancer cells.(10) We hypothesized that distinct co-mutations occurring across cancer lineages might be responsible for the inconsistent responses to FTIs. We further hypothesized that combination therapy targeting the specific co-mutations (MEK inhibitors or PI3K inhibitors) with tipifarnib would improve efficacy.

## Methods

### Genomic Profile by Cancer Type Using Genome Databases

Targeted sequencing data from the MSK-IMPACT(19) and Dana-Farber Cancer Institute (DFCI) cases in the AACR Genomic Evidence Neoplasia Information Exchange (GENIE, Version 9.0)(20) databases were used to investigate co-mutations in *HRAS*, *KRAS* and *NRAS*-mutant salivary, thyroid, bladder, HNSCC, melanoma, prostate, and NSCLC cancers. Canonical oncogenic mutations were selected utilizing OncoKB annotation(21) excluding variants of unknown significance or single nucleotide polymorphisms. Tumors with microsatellite instability or with a tumor mutational burden greater than fifty mutations were excluded. Genetic mutation signatures were grouped by cancer type, and gene mutation frequency compared between *HRAS*, *KRAS* and *NRAS*- mutant tumors.

Co-altered lesions were grouped into three genetic signatures: receptor tyrosine kinase (RTK) pathway mutations, MAP kinase (MAPK) pathway mutations and PI3 kinase (PI3K)/mTOR pathway mutations, and plotted based on cancer type and RAS-mutant type. Specific genes that were categorized are listed in Supplementary Table 1.

Clonality of mutations was assessed using the FACETS (Fraction and Allele-Specific Copy Number Estimates from Tumor Sequencing) algorithm, which provides allele-specific copy number estimates at both the gene level and chromosome arm level. FACETS was used to infer cancer cell fraction associated with individual mutations in order to determine clonality status.(22)

#### Characterization and Transfection of ‘RASless’ Mouse Embryonic Fibroblasts

“Rasless” (*KRAS*^lox/lox^/*NRAS*^*−/−*^/*HRAS*^*−/−*^/*RERT*^*ert/ert*^) mouse embryonic fibroblasts (MEFs) were obtained from the Piro Lito lab (MSKCC, New York, NY) with permission from Dr. Mariano Barbacid (Madrid, Spain).(23) Cells were cultured using a high-glucose DMEM media with penicillin/streptomycin/L-glutamine (PSG; Gemini; #400-110) in 15% fetal bovine serum (FBS). Low passage “Rasless” MEFs were transfected with a HRAS^G13R^ pReceiver-LV205 plasmid (Genecopia) using a lentiviral transfection technique with Opti-MEM, Mission^®^ Lentiviral Packaging Mixture, and Fugune^®^6 Tranfection Reagent (Promega), *Hras*^G13R^-transfected colonies were identified following puromycin selection for at least one week. HRAS-expressing cells were selected and flow-sorted using the GFP signal from the bicistronic vector. Treatment with 600nM tamoxifen (4-OHT) for at least 2 weeks was used to flox-out the KRAS^lox/lox^ allele.

#### Introduction of Co-Altered Mutations to HRAS^G13R^-Transfected MEFs

Co-altered mutations were introduced into “Rasless” MEFs transfected with the HRAS^G13R^ allele. To knock out *Nf1* a dual-guide RNA targeting RAS-binding domain in exon 21 and for *Pten* a dual-guide RNA against phosphatase domain in exon 5 were used. Respective dual-guide RNA with CAS9 and mCherry coexpression plasmids were custom designed and purchased from Vector builder (Supplementary Table 2). CRISPR-CAS9 plasmids were transfected using Fugene, transfected cells were selected by RFP flow sorting, single cell clones isolated, and confirmed for respective CRISPR knock outs. Gain-of-function *Braf*^*G466E*^ (Class 3 BRAF) and *Pik3ca*^*H1047R*^ sequences were cloned into Cs-Mm34053-Lv213 (CMV-mutant gene-IRES2-mCherry-IRES-Blasticidin) (Addgene). They were then introduced using lentiviral transduction with 2 ug/mL blasticidin as the selection antibiotic for at least one week. Mass-transduced cells with the lowest 5% expression levels were selected as determined by flow cytometry using an IRES-mCherry signal as a reporter. Treatment with 4-OHT was used to delete KRAS as described previously(23). *HRAS*^*G13R*^/*Nf1*^*LOF*^ MEFs were not viable when sparcely plated and treated with 4-OHT. As such these cells were kept in highly confluent P10 plates, serially split and continuously treated with tamoxifen for a minimum of 3 weeks.

#### Determination of Cell Viability

Half maximal inhibitory concentration (IC_50_) assays were done by plating on Day 0 either 70,000 cells in 6-well plates in DMEM media with 15% fetal bovine serum (FBS) and penicillin/streptomycin/L-glutamine (P/S/G; Gemini; #400-110) or 100,000 cells for serum-starved (1% FBS) conditions. Cells were treated with the indicated drug concentrations on day 1 with subsequent drug and media change on day 3. Cells were harvested on day 6 by trypsinization and counted using a Vi-Cell series Cell Viability Analyzer (Beckman Coulter). For combination treatments, we plated 3000 cells/well in a 48-well plate in complete media and assayed responses with a crystal violet assay. Cells were treated on Day 1, with drug and media change on day 3. Cells were subsequently fixed and stained with crystal violet on day 6. The following day plates were imaged using Gelcount^™^ analyzer (Oxford Optronics) and subsequently resuspended in 10% acetic acid and counted using a microplate reader (Spectramax^®^ iD5). IC_50_ and crystal violet assays were done in triplicate.

Cell proliferation assays were done by plating 70,000 cells in 6-well plates and treating with either DMSO, monotherapy, or combination therapy for 24h, 48h, 72h, 96h, and 120h. Drug and media change was done at 72h, and cells were harvested at indicated time points. All assays were done in triplicate.

#### Western Blots

Cells treated in the indicated conditions were rinsed with cold PBS and lysed using RIPA (Millipore) supplemented with protease (Complete Mini, Roche) and phosphatase inhibitors (Phosphatase Inhibitor Cocktail Set I and II; Sigma). Protein lysates were collected by spinning cells in a 4-degree centrifuge at 14,000 RPM for 20min. Protein concentrations were measured using the BCA kit (ThemoFisher Scientific) on a microplate reader (SpectraMax^®^ iD5). SDS-PAGE and blocking were done as previously described(24), followed by transfer to a nitrocellulose membrane (0.2mm, Amersham) and blocking in 10% BSA for at least one hour. Membranes were incubated overnight with the indicated primary antibody in 10% BSA in a cold room. Membranes were then washed with Tris-buffered saline with 0.1% Tween^®^ 20 detergent (TBST) and blocked with a Goat anti-Rabbit (IRDye^®^ 680RD) or Goat anti-Mouse (IRDye^®^ 800CW) secondary antibody. Membranes were then imaged using a LI-COR Odyssey^®^ DLx Imaging System.

#### Antibodies

Antibodies used for Western blot analysis included the following: pERK (#9101), total ERK (#4695), pAKT-Ser473 (#9271), pAKT-Thr308 (#13038), total AKT (#2920), PTEN (#9559), PI3K-p110a (#4249) from Cell Signaling; Vinculin (V4139) from Sigma; BRAF (sc-5284) and KRAS (sc-517599) from Santa Cruz; HRAS (18295-1-AP) from ProteinTech; PI3K-p85 (06-195) from Millipore; Total RAS from ThermoScientific (#1862335). All antibodies were diluted to 1:1000.

#### Drugs

Tipifarnib (RR11577), AZD8186 (S7694), Alpelisib (BYL719; S2814), and Pictilisib (GDC-0941, S1065) were purchased from SelleckChem.

#### Statistical Analyses

Genomic analysis of gene signature and gene level mutation frequencies were used to compare co-altered lesion prevalence by cancer type and *RAS*-mutant type. Differences in mutation frequencies between the various cohorts was determined utilizing Fisher’s exact test for binomial proportions. Significance was established as p<0.05.

Statistical analysis for the *in-vitro* studies was performed using GraphPad Prism version 7.0., using two-tailed non-parametric t-tests. Significance was established as p<0.05. IC_50_ curves were generated on GraphPad using non-linear regression.

## Results

### Prevalence of HRAS-mutant tumors amongst cancer types

**Figure 1:**
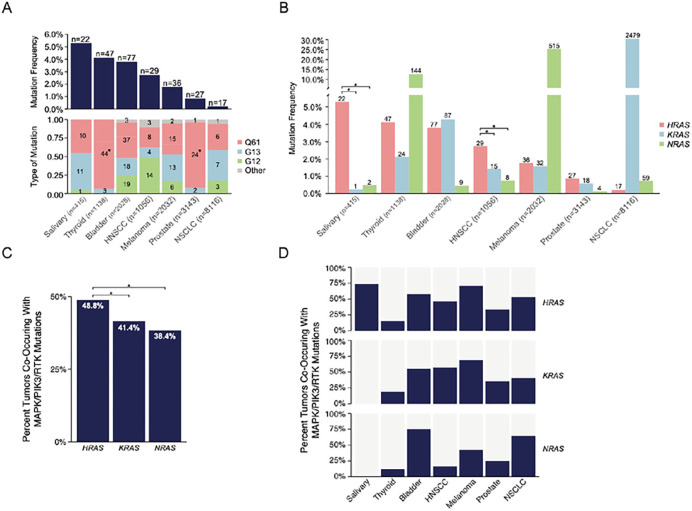
Prevalence of HRAS mutations amongst cancer types. (A) Prevalence of oncogenic *HRAS* mutations among tumors in an institutional cohort (MSK-IMPACT). (B) Percentage of *HRAS*-mutant tumors as compared to those with *KRAS*- and *NRAS* mutations (*p<0.05, Fisher’s Exact Test). (C) Percentage of co-altered mutations in patients with *HRAS*, *KRAS*, or *NRAS*-mutant tumors (*p<0.05, Fisher’s Exact Test). (D) Co-altered mutations partitioned by cancer type.

Altogether 255 (1.4%) patients with canonical *HRAS* mutations were identified in the MSK-IMPACT and DFCI-Genie cohorts, with the number and relative frequency in the evaluated cancer types shown in Figure 1A, top panel. The prevalence of *HRAS* codon mutations differed by cancer type with substitutions at Q61 being the most common, which was particularly enriched in thyroid and prostate cancers (p<0.001 for both) (Figure 1A, bottom).

Overall *KRAS* and *NRAS* were more prevalent than *HRAS* mutations (2516 (14.0%) and 701 (3.9%), respectively; p<0.001 vs *HRAS* for both), as shown in Figure 1B. A greater frequency of *HRAS* mutations were found in salivary gland and HNSCC cancers (both p<0.05). *KRAS* mutations were more prevalent in NSCLC (p<0.01) and *NRAS* mutations in thyroid and melanoma (both p<0.05).

A significantly greater proportion of *HRAS*-mutant cancers (48.4%) were found to have co-altered mutations along the RTK/MAPK/PI3K pathways compared to *KRAS*- (41.4%) and *NRAS*-mutant (38.4%) cancers (Figure 1C) (p<0.05 for both). Among all cancer types, more than half of all *HRAS*-mutant cancers had co-altered lesions, with the exception of those of thyroid and prostate (Figure 1D).

### Co-Altered Mutations in RAS-Mutant Cancers

**Figure 2:**
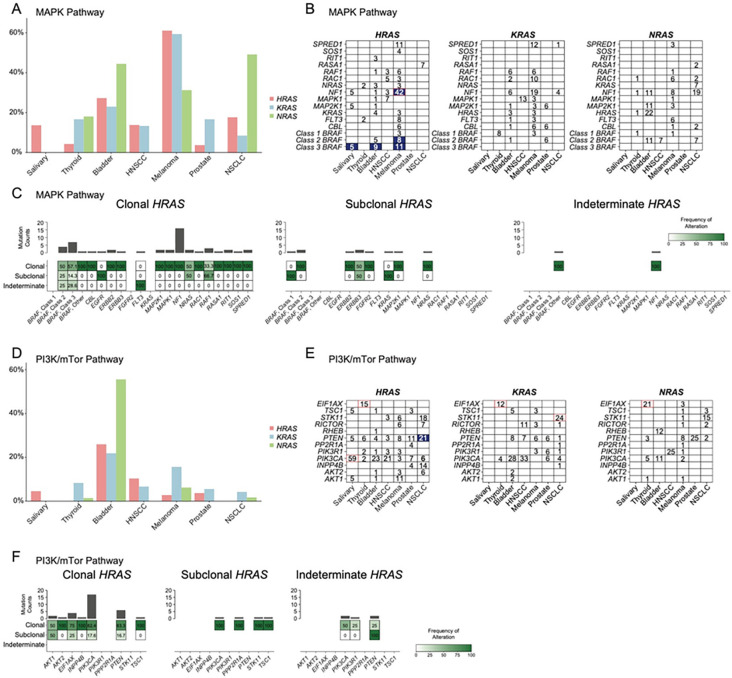
Co-occurring MAPK and PI3K/mTOR pathway mutations in *RAS*mutant tumors. (A). Distribution of MAPK pathway mutations across *HRAS*, *KRAS*, *NRAS* mutant cancers in the MSKCC and DFCI institutional cohorts. (B) Percent frequency of co-occuring mutations in genes encoding MAPK pathway effectors among RAS-mutant cancers (Blue boxes represent significantly found comutation in HRAS-mutant cancer relative to respective *KRAS*- or *NRAS*-mutant cancer [p<0.05]; red box represent significantly found comutation in specific RAS-mutant cancer compared to other cancers with specific RAS mutation [p<0.05])(C) Clonality of co-occuring MAPK pathway mutations in tumors with clonal, subclonal, or indeterminant *HRAS*mutations. D. Distribution of PI3K pathway mutations across *HRAS*, *KRAS*, *NRAS* mutant cancers in the cohorts. (E) Frequencies of co-occuring gene mutations in the PI3K pathway among RAS-mutant cancers. (F) Clonality of co-occuring PI3K pathway mutations in tumors with clonal, subclonal, or indeterminate *HRAS* mutations.

Co-altered mutations prevalent in *HRAS*-, *KRAS*-, and *NRAS*-mutant cancers are outlined in Figure 2. We combined co-altered mutations into signaling pathway categories (MAPK, PI3K/MTOR, and RTK). The prevalence of co-altered mutations in the MAPK pathway in melanomas was the highest among the cancer types we analyzed and was 61.1% for *HRAS*, 59.4% for *KRAS*, and 31.3% for *NRAS*-mutant tumors. In contrast, 27.2% of *HRAS*-mutant bladder cancers carried a co-altered MAPK mutation compared to 23% and 44% of *KRAS* and *NRAS*, respectively (Figure 2A). Amongst *HRAS*-mutant melanomas, 42% carried a co-altered *NF1* mutation, significantly greater than in *KRAS*-/*NRAS*-mutant melanoma and other *HRAS*-mutant cancers (p<0.05). In addition, class 3 *BRAF* mutations were more prevalent in *HRAS*-mutant salivary gland (5%), and bladder (9%), and melanoma (11%) cancers, whereas none were present in the respective *KRAS*- and *NRAS*-mutant tumors (p<0.05 for all). Class 2 *BRAF* mutations were also significantly more prevalent in *HRAS* (8%) compared to *KRAS*- and *NRAS*-mutant melanoma. Clonality analysis of *HRAS*-mutant/MAPK-co-altered tumors was limited to the MSK-IMPACT samples. With few exceptions HRAS mutations were clonal, whereas the clonality of the co-altered lesions was more variable (Figure 2C).

*HRAS*-mutant NSCLC (52.9%) had significantly more co-alterations in PI3K/mTOR pathway genes compared to *KRAS* (32.2%) and *NRAS* (23.7%) (Figure 2D; p<0.05). This was true for *PTEN* (21%) (p<0.05 vs *KRAS*- and *NRAS*-mutant NSCLC). *PIK3CA* mutations were found in 59% of *HRAS*-mutant salivary gland tumors but due to the limited number of *KRAS*- and *NRAS*-mutant tumors of this lineage this was not significantly different, but was more prevalent than in other *HRAS*-mutant cancers (p<0.05). Of note, *EIF1AX* mutations were found almost exclusively in *HRAS*-, *KRAS*-, and *NRAS*-mutant thyroid cancers (p<0.05), whereas *STK11* mutations were found in greater proportion of *HRAS*-mutant NSCLC compared to the other *HRAS*-mutant cancer types. Clonality analysis for *HRAS*-mutant/*PI3K*-co-altered tumors and noteworthy for the majority of *HRAS*-mutant/*PIK3CA* (82.4%) and *PTEN*-mutant tumors (83.3%) being clonal (Figure 2F).

### Targeting HRAS-Mutations in “Rasless” MEFs

**Figure 3:**
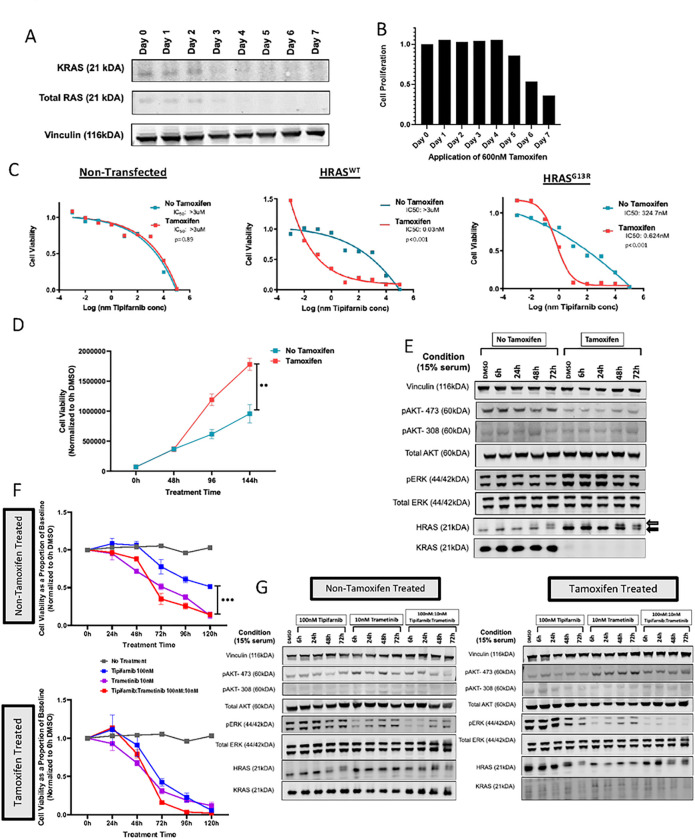
HRAS^G13R^ sensitizes RASless mouse embryonic fibroblasts (MEFs) to tipifarnib. (A) Western blot and (B) proliferation of “RASless” (KRAS^lox^/HRAS^KO^/NRAS^KO^) MEFs exposed to 600nM Tamoxifen (4-OHT) over 7 days. (C) Tipifarnib IC50 curves in non-transfected, HRAS-WT/HRAS^G13R^ - transfected RASless MEFs exposed to 4-OHT or vehicle (p value based on triplicate IC50 values, t-test). (D) (D) Proliferation of HRAS^G13R^ - transfected RASless MEFs with and without 4-OHT. (E) Western blot of RASless MEFs treated with 100nM tipifarnib over 72 hours with and without 600nM 4-OHT. Arrows indicate molecular weight shift of defarneylsated HRAS with tipfarnib exposure. (F) Cell viability of HRAS^G13R^ - transfected RASless MEFs with (top panel) and without 4-OHT (bottom panel). Cells were treated with tipifarnib, trametinib or the combination (t-test, ***p<0.001: tipifarnib vs trametinib, tipifarnib vs tipifarnib/trametinib). (G) Westernblot of the same cells as in (F) collected up to 72 hours.

“Rasless” (*Kras*^lox/lox^;*Nras*^*−/−*^;*Hras*^*−/−*^;*RERT*^ert/ert^) MEFs were utilized to characterize the growth, signaling, and drug sensitivity of the most common co-altered mutations identified in the MSK-IMPACT and GENIE data (Figure 3). We confirmed loss of *Kras*^WT^ expression when MEFs were exposed to 600nM tamoxifen (4OHT).(25) Loss of *Kras* and total *Ras* was apparent by Day 3–4 of treatment (Figure 3A), with a decrease in cell proliferation by Day 4–5 (Figure 3B). Rasless MEFs were resistant to tipifarnib in both the tamoxifen- and non-tamoxifen-treated setting (IC_50_: >3uM for both) (Figure 3C, left).

Introduction of an *Hras*^WT^ allele did not confer sensitivity to tipifarnib in the presence of a *Kras*^WT^ allele (IC50: >3uM) but demonstrated exquisite sensitivity in the presence of tamoxifen (IC_50_: 0.03nM, p<0.001), consistent with the on-target effect of the FTI (Figure 3C, middle). By contrast expression of Hras^G13R^ (Figure 3C, right) partially sensitized cells to tipifarnib (IC_50_: 324.7nM) as compared to parental and Hras^WT^-transfected cells even in the presence of *Kras*^WT^ (p<0.001), whereas tamoxifen-induced *Kras*^WT^ loss rendered Hras^G13R^-expressing cells highly sensitive to treatment (IC50: 0.6nM; p<0.001).

Tamoxifen-treated *Hras*^*G13R*^-transfected cells expanded more rapidly after 6 days (1.8 ± 0.1 × 10^6^ vs. 1.0 ± 0.2 × 10^6^ cells; p<0.01), suggesting that *Kras*^WT^ expression had a dampening effect on the proliferative drive induced by mutant Hras (Figure 3D). A time course of the effects of tipifarnib on signaling in *Hras*^G13R^-transfected cells treated without or with 4OHT is shown in Figure 1E. HRAS defarnesylation (clear arrow) was detectable 48–72h after exposure to tipifarnib regardless of 4OHT treatment. Baseline *HRAS* and pERK levels were higher in MEFs lacking *Kras*^WT^ consistent with its inhibitory effects on *Hras*^G13R^-induced cell growth, and were inhibited by tipifarnib, whereas pERK levels were not affected by tipifarnib in the absence of 4OHT. Taken together, expression of *Kras*^*WT*^ attenuates the MAPK signaling and the proliferative drive induced by *Hras*^G13R^, and renders cells less sensitive to tipifarnib.

We next investigated whether the dampening effect of *Kras*^WT^ on sensitivity to tipifarnib was due to unrestrained MAPK signaling output, particularly in the presence of serum. *Hras*^G13R^- mutant MEFs were highly sensitive to the MEK inhibitor trametinib (non-4OHT IC_50_: 5.7nM; 4OHT IC_50_: 0.9nM) (Supplementary Figure 1A,B). Crystal violet assays were performed to identify appropriate combination doses of tipifarnib and trametinib, with 100nM:10nM tipifarnib:trametinib determined to adequately inhibit growth in non-4OHT-treated *Hras*^G13R^- mutant MEFs (Supplementary Figure 1A,B). Trametinib and combination tipifarnib:trametinib treatment were highly effective at treating *Kras*^WT^-expressing *Hras*^G13R^ cells, with cell viability relative to DMSO significantly lower than tipifarnib monotherapy at 120h (tipifarnib: 0.5 ± 0.02; trametinib:tipifarnib: 0.1 ± 0.02; p<0.001) (Figure 3F). In 4OHT-treated cells, combination trametinib:tipifarnib decreased viability as compared to tipifarnib at 72h (tipifarnib: 0.4 ± 0.05; trametinib:tipifarnib: 0.2 ± 0.01; p<0.001), but the effect was lost at 120h (tipifarnib: 0.06 ± 0.02; trametinib:tipifarnib: 0.02 ± 0.02; p=0.08). Levels of pERK were inhibited at 6h in both conditions (Figure 3G), but rebounded in *Kras*^WT^-expressing cells with trametinib monotherapy and tipifarnib:trametinib combination. The pERK rebound with combination tipifarnib:trametinib abated in 4OHT-treated cells.

### Introduction of Co-Altered Mutations Leads to Resistance to Tipifarnib:

#### Class 3 *BRAF* (*Hras*^G13R^/*Braf*^G466E^)

**Figure 4:**
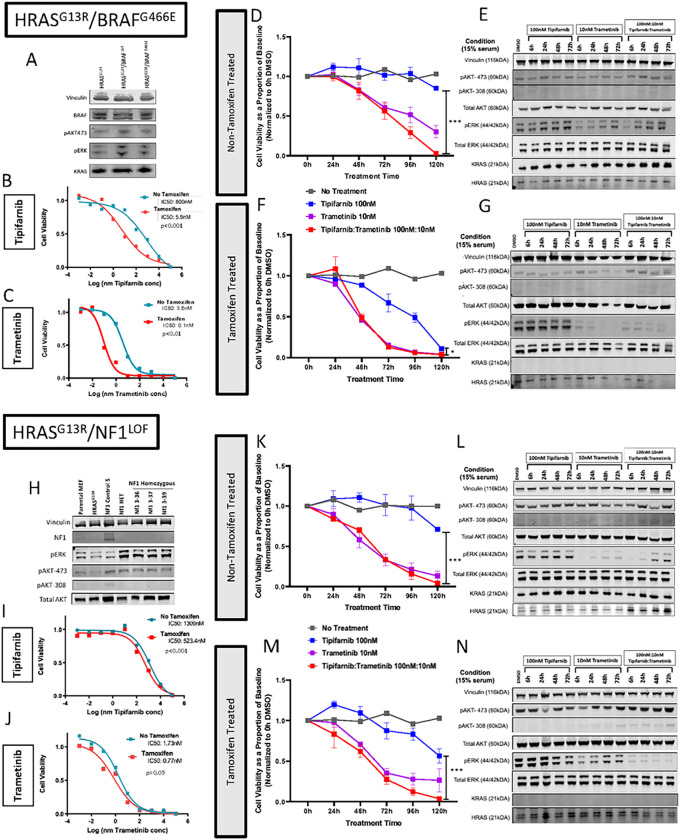
Class 3 BRAF (G466E) and NF1 loss of function mutations result in resistance to tipifarnib in RASless MEFs with HRAS^G13R^. (A) HRAS^G13R^ MEFs were transfected with BRAF^G466E^ or BRAF^WT^. Western blot demonstrates relative expression of BRAF in non-transfected and transfected cells. (B) IC50 curves for HRAS^G13R^/BRAF^G466E^ MEFs exposed to tipifarnib with and without 4-OHT (p value based on triplicate IC50 values, t-test). (C) IC50 curves for HRAS^G13R^/BRAF^G466E^ MEFs exposed to trametinib with and without 4-OHT (p value based on triplicate IC50 values, t-test). (D, E) Cell viability of HRAS^G13R^/BRAF^G466E^ MEFs with and without 4-OHT. Cells were treated with tipifarnib, trametinib or the combination (t-test, ***p<0.001, * p<0.05). (F, G) Western blot of HRAS^G13R^/BRAF^G466E^ MEFs treated with indicated drugs up to 72 hours. (H) Western blot of HRAS^G13R^ MEFs that underwent sequential rounds of CRISPR-Cas9 to induce homozygous loss of NF1. (I,J) IC50 curves for HRAS^G13R^/NF1^lof^ exposed to tipifarnib and trametinib with and without 4-OHT (p value based on triplicate IC50 values, t-test). (K,L) Cell viability of HRAS^G13R^/NF1^lof^ MEFs with and without 4-OHT. Cells were treated with tipifarnib, trametinib or the combination (t-test, ***p<0.001:, tipifarnib vs tipifarnib/trametinib). (F, G) Western blot of HRAS^G13R^/NF1^lof^ MEFs treated with indicated drugs up to 72 hours.

Following transduction of bicistronic lentiviral vectors for *Braf*^WT^ or *Braf*^G466E^ cDNA into *Hras*^G13R^-expressing MEFs, cells were sorted for mCherry to select those in the 5^th^ percentile of expression, which mimicked endogenous levels of *Braf* (Figure 4A). Sensitivity to tipifarnib was decreased in Braf^G466E^ compared to *Braf*^WT^-expressing cells in the absence of 4OHT (IC50 *Braf*^G466E^ vs *Braf*^WT^: 800nM vs 161.9nM; p<0.05), whereas in 4OHT-treated cells they were highly sensitive to tipifarnib and not significantly different to each other (IC50 *Braf*^G466E^ vs *Braf*^WT^: 5.9nM vs 6.9nM) (Supplementary Figure 2). Trametinib monotherapy effectively inhibited the growth of *Hras*^G13R^/*Braf*^G466E^ cells (Figure 4C), with 4OHT-treated cells demonstrated greater sensitivity than non 4OHT-treated cells (0.1nM v 3.8nM, respectively; p<0.01).

Resistance of *Hras*^G13R^/*Braf*^G466E^ cells to the growth inhibitory effects of tipifarnib was relieved by the combination with trametinib in cells with *Kras*^WT^ expression, with the combination also being superior to trametinib alone (Figure 4D). Addition of trametinib significantly reduced pERK after 6h, although rebound was observed with trametinib and combination tipifarnib:trametinib therapy (Figure 4E). 4OHT-treatment partially sensitized cells to tipifarnib. Addition of trametinib inhibited their growth more profoundly, but the effects of the combination were not superior to trametinib monotherapy (Figure 4F).

Tamoxifen-treated cells (Figure 4F) cell viability demonstrated sensitivity to tipifarnib therapy by 120hrs however was still significantly reduced compared to combination 100nM:10nM tipifarnib:trametininb treatment (tipifarnib:trametininb- 0.04 ± 0.01 vs tipifarnib- 0.11 ± 0.01; p<0.001). In addition, trametinib demonstrated improved reduction in cell viability at all time points leading up to 120h. The deletion of *Kras*^WT^ by the addition of 4OHT eliminated the late pErk rebound observed after treatment with trametinib (Figure 4G).

#### *NF1* Loss-Of-Function (*Hras*^G13R^/*Nf1*^LOF^)

To study *NF1* loss, sgRNAs targeting exon 21 of *Nf1* were designed to create loss-of-function mutations in *Hras*^G13R^-transfected cells. Two rounds of CRISPR-Cas9 were required to first generate a heterozygous clone and subsequently cells with homozygous *Nf1* loss (Suppl Fig 3). Signalling was performed amongst various suspected homozygous clones with demonstrated increased pERK signal with NF1 loss-of-function (Figure 4H). Clone 39, hereafter designated as *HRAS*^G13R^*/Nf1*^LOF^, was used for analysis.

The introduction of an *Nf1*^LOF^ homozygous mutation resulted in resistance to tipifarnib but retained sensitivity to trametinib (non-4OHT IC_50_: 1.73nM vs 4OHT IC_50_: 0.8nM) (Figures 4I and 4J), with consistent findings in the cell viability and signalling assays (Figure 4K–N). Interestingly, the rebound of pERK after trametinib treatment was still present in *Hras*^G13R^/*Nf1*^LOF^ cells treated with 4OHT. This is consistent with a loss of an inhibitory effect of *Nf1* on mutant *Hras*, since the rebound is relieved by the combination of tipifarnib and trametinib.

#### *Pten* Loss-of-Function (*Hras*^G13R^/*Pten*^LOF^)

**Figure 5:**
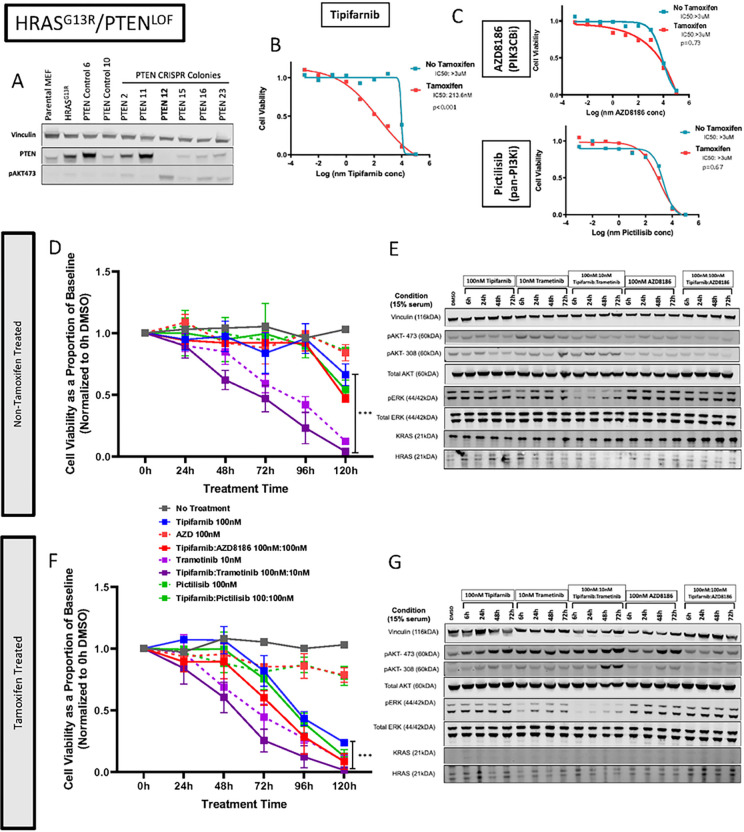
PTEN loss-of-function mutation results in tipifarnib resistance in RASless MEFs with HRAS^G13R^. (A) Western blot of HRAS^G13R^ MEFs after CRISPR-Cas9 introduced a PTEN^LOF^ mutation. (B) IC50 curves of HRAS^G13R^/PTEN^lof^ MEFs exposed to tipifarnib with and without 4-OHT (p value based on triplicate IC50 values, t-test). (C) IC50 curves of HRAS^G13R^/PTEN^lof^ MEFs exposed to AZD8186 (top panel) and pictilisib (lower panel). (D,E) Cell viability of HRAS^G13R^/PTEN^lof^ MEFs with and without 4-OHT. Cells were exposed to tipifarnib, trametinib, AZD8186, Pictilisib or combinations (t-test, ***p<0.001:, tipifarnib vs tipifarnib/trametinib). (F, G) Western blot of HRAS^G13R^/PTEN^lof^ MEFs treated with indicated drugs up to 72 hours.

We generated *Pten* loss-of-function mutations in *Hras*^G13R^-transfected MEFs using sgRNA targeting of exon 5. Western blot analysis identified one clone with a homozygous *Pten* loss-of-function mutation and we observed concomitant increased pAkt-Ser473 signal (Figure 5A). At baseline *Hras*^G13R^/*Pten*^LOF^ cells were resistant to monotherapy with tipifarnib, which was partially relieved by 4OHT. Cells were insensitive to AZD-8186 (PI3KCB-inhibitor) or Pictilisib (pan-PI3K inhibitor) monotherapy in the absence or presence of 4OHT (Figures 5B and 5C). Crystal violet assays were also used to select an optimal combination drug combination to test cell viability in *Hras*^G13R^/*Pten*^LOF^ cells (Supplementary Figure 4) which demonstrated relative resistance to combination PI3K inhibition.

In the absence of 4OHT, growth assays of *Hras*^G13R^/*Pten*^LOF^ MEFs was insensitive to the tipifarnib:AZD8186 or tipifarnib:pictilisib combinations (Figure 5D–E). When exposed to 4OHT, there was modest additive growth inhibitory effects of combination tipifarnib:AZD8186 therapy (Figure 5F–G). Western blots of Hras^G13R^/Pten^LOF^ cells treated with AZD8186 or tipifarnib:AZD8186 showed reduction in pAkt-Ser473 and pAkt-Thr308, consistent with on target effects of the PI3KCB-inhibitor. This was also true for pictilisib, particularly in the absence of 4OHT (Supplementary Figure 5).

Next, we tested the combination of tipifarnib/MEK inhibitor in *Hras*^G13R^/*Pten*^LOF^ cells given the sensitivity we observed in *Hras*^G13R^, *Hras*^G13R^/*Braf*^G466E^, and *Hras*^G13R^/*Nf1*^lof^ Rasless-MEFs. Crystal violet assays were again employed to define optimal concentrations of drug (Supplementary Figure 4). Despite the loss of function of *Pten*, trametinib monotherapy and combination tipifarnib:trametinib had significant anti- proliferative effects that were augmented by 4OHT (Figure 5F). Interestingly, trametinib was ineffective in inhibiting ERK phosphorylation in cells expressing wild type *Kras*. Treatment with 4OHT was associated with transient pERK inhibition by the MEK inhibitor, whereas the combination of tipifarnib and trametinib induced sustained MAPK inhibition, which was more accentuated in *Kras*-deficient cells. Akt phosphorylation was not inhibited by trametinib or trametinib:tipifarnib combination therapy. Hence, upon loss of *Pten*, *Hras*-mutant cells develop resistance to tipifarnib, which is relieved by MAPK pathway inhibition, but not by blocking PI3K signaling.

#### *Pik3ca*^H1047R^ Gain of Function (*Hras*^G13R^/*Pik3ca*^H1047R^)

**Figure 6:**
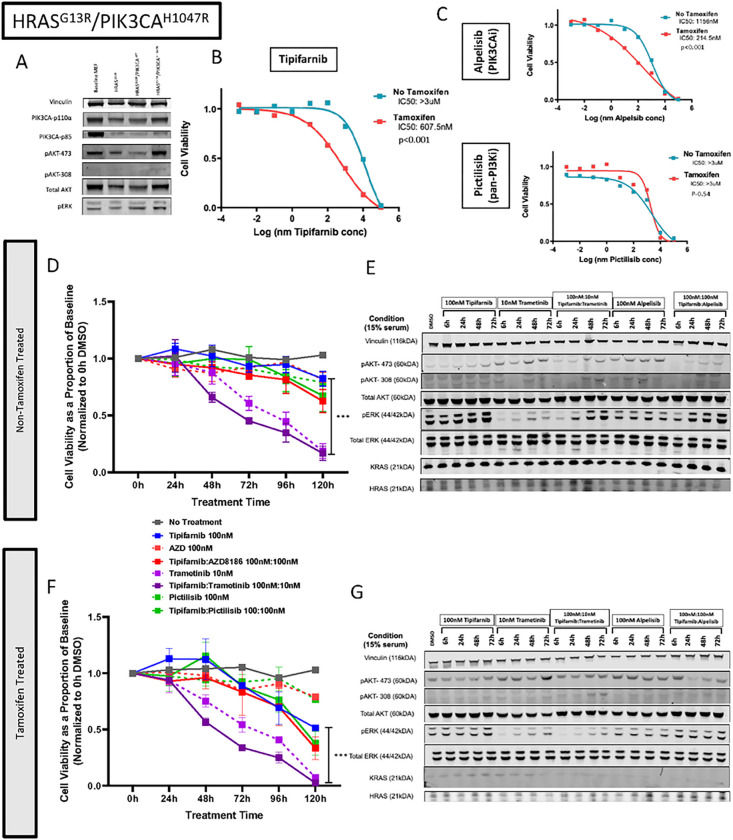
PIK3CA^H1047R^ mutation results in tipifarnib resistance. (A) Western blot of HRAS^G13R^ MEFs transduced with vectors carrying PIK3CA^H1047R^ and PIK3CA^WT^.(B) IC50 curves of HRAS^G13R^/ PIK3CA^H1047R^ MEFs exposed to tipifarnib with and without 4-OHT (p value based on triplicate IC50 values, t-test). (C) IC50 curves of HRAS^G13R^/ PIK3CA^H1047R^ MEFs exposed to AZD8186 (top panel) and pictilisib (lower panel). (D,E) Cell viability of HRAS^G13R^/ PIK3CA^H1047R^ MEFs with and without 4-OHT. Cells were exposed to tipifarnib, trametinib, AZD8186, Pictilisib or combinations (t-test, ***p<0.001:, tipifarnib vs tipifarnib/trametinib). (F, G) Western blot of HRAS^G13R^/ PIK3CA^H1047R^ MEFs treated with indicated drugs up to 72 hours. (H, I) Cell proliferation of HRAS^G13R^ MEFs co-altered with indicated mutations with and without 4-OHT in high (15%) and low (1%) serum conditions.

*Pik3ca*^H1047R^ mutations were introduced into *Hras*^G13R^-transfected MEFs. To mimick endogenous levels of oncoprotein, we selected the lowest 5% of vector-expressing cells using an IRES-mCherry signal for both Pik3ca^WT^ and Pik3ca^H1047R^. Western bloting demonstrated similar PI3K-p110a and PI3K-p85 levels between the Hras^G13R^, Hras^G13R^/Pik3ca^WT^, and Hras^G13R^/Pik3ca^H1047R^ cells with pAkt-Ser473 signal increased in the latter (Figure 6A). Similar to Hras^G13R^/Pten^LOF^ cells, Hras^G13R^/Pik3ca^H1047R^ cells demonstrated resistance to tipifarnib, alpelisib (PIK3CA-inhibitor) and picitilisib monotherapies (Figures 6B and 6C).

As in previous experiments, we identified sensitive concentrations of drugs using crystal violet assays (Supplementary Figure 6). Resistance to tipifarnib, alpelisib, and picitilisb monotherapy was not significantly overcome with combination tipifarnib:alpelisib or tipifarnib:pictilisib therapy (Figure 6D–E). However, treatment with combination tipifarnib:trametinib significantly improved sensitivity, even more so in presence of 4OHT, associated with pERK inhibition (Figure 6F,G). Signaling responses to pictilisib:tipifarnib were similar to those of tipifarnib:alpelisib (Supplementary Figure 7). Baseline *Hras*^G13R^ MEFs also conferred no added sensitivity with combination tipifarnib:AZD8186 and tipifarnib:alpelisib (Supplementary Figure 8)

### Endogenous KRAS expression slows proliferation in HRAS^G13R^-transfected MEFs

**Figure 7:**
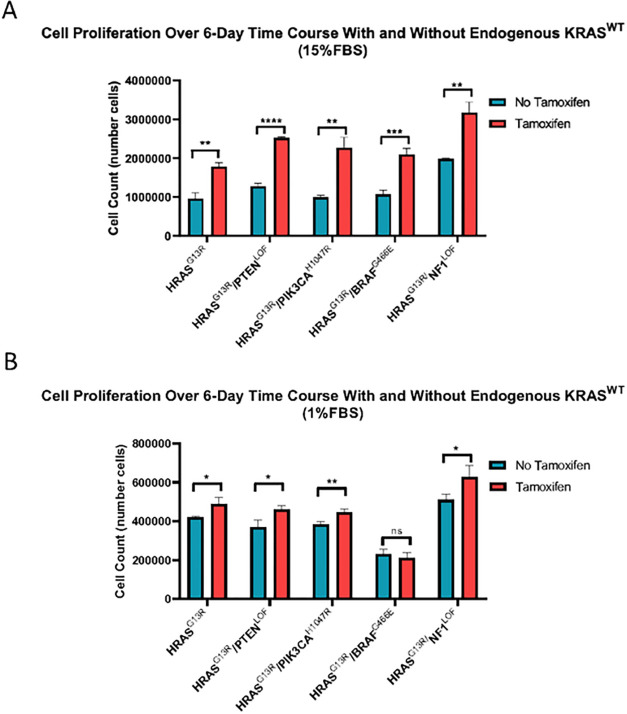
Cell proliferation in HRAS^G13R^-transfected MEFs is slowed in the preszence of endogenous KRAS^WT^. Cell proliferation after 6 days of untreated growth were tested in (A) 15% serum and (B) 1% serum conditions. HRAS^G13R^-transfected cells were tested along with indicated co-mutations witht and without 4-OHT in both high and low serum conditions. (t-test, *p<0.05, **p<0.01, ***p<0.001, ****p<0.0001, ns=not significant).

We assessed the effect of endogenous *KRAS* expression on proliferation of *Hras*^G13R^-transfected MEFs with or without co-mutations. Cells were grown for 6 days in 15% (Figure 7A) or 1% FBS (Figure 7B). Interestingly, *Kras*^WT^ suppressed cell proliferation in 15% serum across all cell conditions, an effect that was partially relieved in *Hras*^G13R/^*Nf1*^LOF^ cells. *Kras*^WT^ knockdown with 4OHT derepressed cell growth in all the genetic contexts. These effects were serum-dependent, since in 1% serum growth of MEFs was unaffected by the presence or absence of *Kras*^WT^.

## Discussion

Tipifarnib, a farnesyltransferase inhibitor, impedes the activation of oncogenic *HRAS* by preventing its translocation to the plasma membrane and subsequent downstream effector signaling.(14) Using targeted exome sequencing data we show that *HRAS*-mutant cancers are associated more commonly with co-mutations of genes encoding effectors in the MAPK and PI3K pathway than tumors driven by *KRAS* or *NRAS*, with specific lineage differences. We used “RASless” MEF lines expressing mutant *HRAS* to investigate whether some of the more common co-mutations observed in human tumors affected their response to growth inhibition by tipifarnib, which is currently in clinical development for *HRAS*-mutant cancers. We found that concurrent mutations of *PTEN, PIK3CA*, *NF1,* or a class 3 *BRAF* mutation reduce the efficacy of tipifarnib. Regardless of whether the co-mutant protein canonically signals in the PI3K or MAPK pathway, combination treatment with a MEK inhibitor, but not with pan-PI3K or isoform selective PI3K inhibitors, enhances sensitivity to the FTI.

The presence of a wild-type *KRAS* allele attenuated serum-induced growth of the *HRAS*^*G13R*^-expressing MEFs. It is well established that *Hras*-*WT* dampens transformation by mutant *HRAS* in mouse models. (26) This is also true for *Kras*-*WT* in KRAS-mutant tumors, and for *Nras*-*WT* in *NRAS*-mutant tumors.(27) Moreover, allelic imbalance, often due to LOH of the corresponding wild-type alleles, is commonly present in oncogenic RAS-driven cancers of different lineages and associated with disease progression, (27) whereby wild-type RAS alleles reduce the activation threshold of mutant RAS alleles.(28, 29) The inhibitory effect of wild-type *KRAS* on oncogenic *KRAS*-induced growth has been attributed to homodimerization, since introduction of a mutation that impairs *KRAS* homodimer formation without affecting its effector function abrogates its growth inhibitory effects.(30) The crystal structure of *HRAS* identified a potential dimer interface similar to that of *KRAS*,(31) but it is unclear whether *HRAS* can heterodimerize with other RAS family proteins, or whether *HRAS*-WT dampens the growth promoting effects of *HRAS*^G13R^ through alternative mechanisms.

Endogenously expressed wild-type *Kras* imparted resistance to FTI therapy in *Hras*^G13R^-transfected MEFs. Expression of wild-type *RAS* has been shown to contribute to adaptive resistance to therapeutic targeting of mutant *BRAF* or *MEK*.(27, 32, 33) In our models, wild-type *KRAS* decreased sensitivity of *HRAS*^G13R^-MEFs to tipifarnib even when co-altered mutations in the MAPK and PI3K pathway were introduced. These findings suggest that allelic balance of wild type RAS proteins likely plays a role in response to tipifarnib therapy. The ratio of wild-type:mutant *RAS* alleles requires further study as a biomarker for FTI sensitivity of *HRAS*-mutant cancers.

Wild-type RAS proteins play an important role in determining rebound activation of the MAPK pathway that imparts resistance to targeted therapies.(10, 34) In our study, trametinib induced rebound pERK signal by 48–72hrs in cells with any co-altered mutations in the presence of wild type *Kras*. We also noted rebound signaling of pAkt in the *Hras*^G13R^/*ras*^LOF^ and *Hras*^G13R^/*Pik3ca*^H1047R^ cells. In the presence of tipifarnib and trametinib, cells that continued to express wild-type *KRAS* had similar rebound signaling that was abated when cells were treated with 4OHT. This is consistent with data demonstrating combination treatment targeting downstream MAPK signaling and upstream RTKs or *SHP2/SOS* is an effective anti-tumor strategy in *RAS* mutant tumors. (34–37)

We identified co-mutations in MAPK and PI3K pathway genes that, in the setting of mutant *HRAS*, imparted resistance to FTI therapy. Class 3 *BRAF* mutations and *NF1* loss were common in various *HRAS*-mutant cancers, in particular melanomas. Loss-of function mutations to *NF1*, a RAS GTPase activating protein, confer resistance to tyrosine kinase inhibitors and targeted therapies across tumor types.(38–40) We previously showed that knock-down of *Nf1* in murine *Hras*-mutant poorly differentiated thyroid cancer cell lines imparted resistance to FTI therapy via feedback activation of wild type *RAS* proteins, which required *MEK* inhibition to abate rebound signaling and enhance growth inhibition.(10) CRISPR-Cas9 knockout of *NF1* also caused profound resistance to tipifarnib in *HRAS*^G13R^ MEF, which was only partially abrogated by *KRAS* deletion. This latter finding is intriguing, as it could imply that loss of NF1 either further enhances mutant *HRAS* activity, or is acting in a *RAS*-independent manner in the otherwise RASless cells.

Class 3 *BRAF* mutations are insensitive to drugs that target class 1 BRAF mutant proteins that signal as monomers, such as dabrafenib or vemurafenib(41, 42). They represent a subset of *BRAF* mutant proteins that heterodimerize with CRAF and are RAS-dependent for activation.(43) As a result, we hypothesized that a class 3 *BRAF* co-mutation in *HRAS*^*G13R*^-MEFs would render cells sensitive to tipifarnib treatment. This was true when *Kras*-*WT* was deleted, but not when *KRAS* was present, consistent with the dependence of *BRAF*^G466E^ on *RAS* signaling.

We found that comutations in the PI3K/mTOR pathway also imparted resistance to FTIs. Mutations in the PI3K pathway have been reported in patients with *KRAS*^*G12C*^-mutant tumors who developed resistance to the *KRAS*^G12C^ inhibitor adagarsib in the KRYSTAL-1 trial.(44) Combination tipifarnib and the *PI3KA* inhibitor alpelisib is currently being used in a Phase II trial to target *HRAS*-mutant tumors with *PIK3CA* mutations (KURRENT-HN trial; NCT04809233). We found the combination of tipifarnib and alpelisib to have limited efficacy in the *Hras*^*G13R*^/*Pik3ca*^*H1047R*^ MEFs, which was modestly improved by deletion of *Kras*-*WT*. Instead the combination of the *MEK* inhibitor trametinib and tipifarnib was highly effective for *Hras*^*G13R*^ MEFs harboring either *Pik3ca*^*H1047R*^ or deletion of *Pten*. Interestingly, *Pten* loss enhances MAPK activation and *MEK* inhibitor sensitivity in a mouse model of Her2/neu-driven breast cancer.(45) These findings indicate that persistent activation of MAPK is a dominant mechanism of resistance to FTIs even in the presence of PI3K pathway activation. An important caveat to our findingsis is that the experiments were conducted in mouse embryonic fibroblasts, which may not fully reflect cell lineage-specific signaling or therapeutic dependencies.

In summary, results from our study highlight the importance of co-altered mutations and of wild-type RAS in driving resistance to targeted therapy of *HRAS*-mutant cancers. The combination of FT and MEK inhibitors was efficacious regardless of co-mutation status, which may help inform the design of future clinical trials.

## Data Availability

The datasets generated during and/or analysed during the current study are available from the corresponding author on reasonable request.
